# Metastatic progression is associated with dynamic changes in the local microenvironment

**DOI:** 10.1038/ncomms12819

**Published:** 2016-09-15

**Authors:** Nicole M. Aiello, David L. Bajor, Robert J. Norgard, Amine Sahmoud, Neha Bhagwat, Minh N. Pham, Toby C. Cornish, Christine A. Iacobuzio-Donahue, Robert H. Vonderheide, Ben Z. Stanger

**Affiliations:** 1 Abramson Family Cancer Research Institute, Perelman School of Medicine, University of Pennsylvania, Philadelphia, Pennsylvania 19104, USA; 2 Department of Pathology, Johns Hopkins University, Baltimore, Maryland 21231, USA

## Abstract

Most cancer-associated deaths result from metastasis. However, it remains unknown whether the size, microenvironment or other features of a metastatic lesion dictate its behaviour or determine the efficacy of chemotherapy in the adjuvant (micrometastatic) setting. Here we delineate the natural history of metastasis in an autochthonous model of pancreatic ductal adenocarcinoma (PDAC), using lineage tracing to examine the evolution of disseminated cancer cells and their associated microenvironment. With increasing size, lesions shift from mesenchymal to epithelial histology, become hypovascular and accumulate a desmoplastic stroma, ultimately recapitulating the primary tumours from which they arose. Moreover, treatment with gemcitabine and nab-paclitaxel significantly reduces the overall number of metastases by inducing cell death in lesions of all sizes, challenging the paradigm that PDAC stroma imposes a critical barrier to drug delivery. These results illuminate the cellular dynamics of metastatic progression and suggest that adjuvant chemotherapy affords a survival benefit by directly targeting micrometastases.

## Abstract (web summary)

Metastatic colonization—the growth of single metastatic cells or small lesions into gross metastases—is poorly understood. Here, the authors characterize the natural history of metastatic growth and assess the impact of chemotherapy in an autochthonous model of pancreatic cancer.

## Graphical abstract

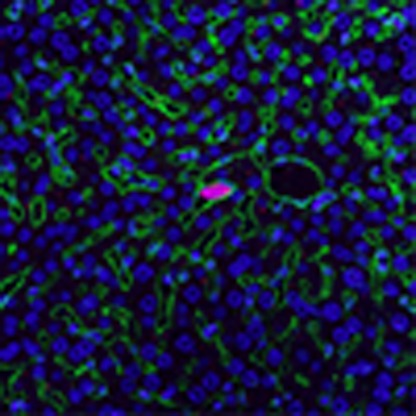

Pancreatic ductal adenocarcinoma (PDAC) has a 5-year survival rate of less than 6%, a dismal outcome related to late detection and a high rate of spread at the time of diagnosis^1^,^2^. Hence, metastatic disease accounts for a majority of PDAC-related deaths, even for patients with resectable tumours and no evidence of metastasis at the time of diagnosis^3^,^4^. However, the mechanisms by which tumour cells navigate the ‘metastatic cascade'—a gauntlet that requires cellular escape from the primary tumour, survival in the circulation, invasion into distal tissues and colonization (growth) in a foreign, potentially hostile microenvironment—remain poorly understood^5^.

One process thought to facilitate metastasis is epithelial-mesenchymal transition (EMT), whereby epithelial cells lose their adhesive contacts with neighbours and take on the migratory phenotype of mesenchymal cells^6^. Although EMT is believed to play a role in the dissemination of carcinoma cells, it has also been observed that metastases tend to exhibit the epithelial histology of their parent primary tumours^7^,^8^,^9^. This has led to the idea that the reverse process—mesenchymal-epithelial transition (MET)—drives the formation of a more epithelial phenotype at metastatic sites^10^. Although several studies support the view that MET is critical for metastatic colonization^11^,^12^,^13^, evidence for this phenomenon is lacking from spontaneous tumour models.

Like primary tumours, metastases are a conglomerate of cancer cells, stromal cells and extracellular matrix (ECM). In primary pancreatic cancer, a particularly dense (desmoplastic) stroma containing leukocytes, fibroblasts and ECM makes up a large portion of the tumour mass, while the density of blood vessels tends to be low^14^. Although macro-metastatic lesions of various cancers—including PDAC, ovarian and breast—also exhibit a dense stromal infiltrate, it remains unclear when during metastatic progression this re-establishment of the tumour microenvironment occurs.

Here, we have taken advantage of a lineage-labelled autochthonous model of pancreatic cancer to carefully catalogue the changes that occur within metastatic cells and their immediate microenvironment during metastatic colonization and growth. We report that the process is highly dynamic, as both tumour cells and the stroma undergo marked changes during progression from singly seeded cells to micro- and macro-metastases. Furthermore, we show that treatment with gemcitabine and nab-paclitaxel results in killing of metastatic tumour cells despite the presence of an ostensibly protective microenvironment. These results provide insight into the process of metastatic colonization and challenge the hypothesis that stroma acts as a physical barrier to drug delivery.

## Results

### Lineage tracing permits detection of micro-metastases

**Figure 1:**
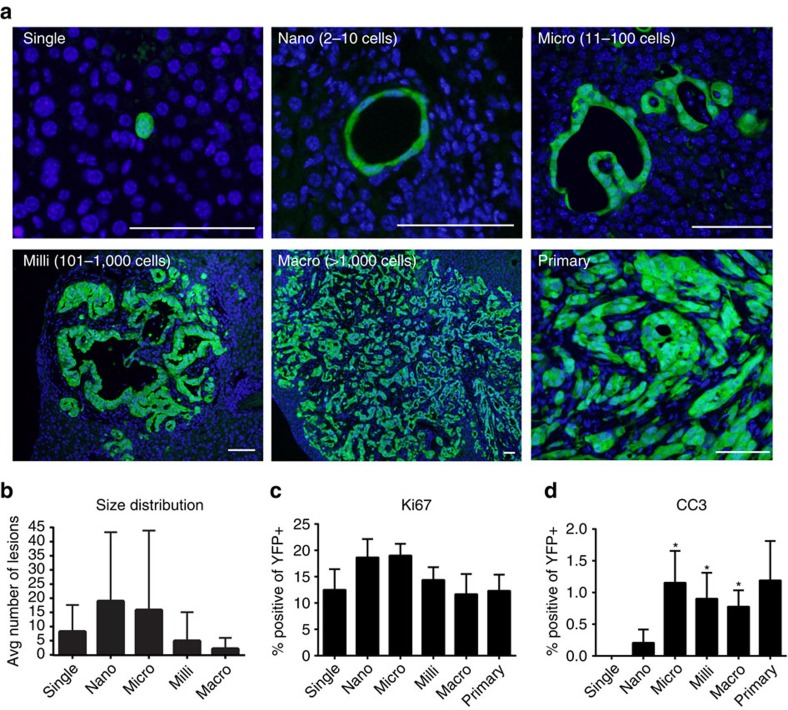
Metastatic landscape of the KPCY model. (**a**) Representative images of YFP^+^ liver metastases and primary tumour. Scale bars, 50 μm. (**b**) Size distribution of metastatic lesions in the liver grouped according to size (*n*=23 mice). (**c**) Quantification of KI67^+^ (*P*=0.9439) tumour cells in liver metastases (*n*=6 mice, 159 lesions). (**d**) Quantification of CC3^+^ (*P*=0.1621) tumour cells in liver metastases (*n*=4 mice, 46 lesions). *P*-values were calculated by one-sample *t*-test against ‘single cell' means. Data are presented as mean±s.d.

We began by characterizing metastatic PDAC lesions in KPCY mice—in which **C**re-mediated recombination triggers mis-expression of mutant **K**ras^G12D^, deletion of one copy of **p**53, and activation of a **Y**FP lineage label in pancreatic epithelial cells—allowing us to track tumour cells at the single cell level^15^. KPCY mice, like related mouse models^16^,^17^, exhibit a pattern of gross metastasis similar to the human disease, consisting of spread to liver (40.5% of mice), diaphragm (32.4%), lung (10.8%), peritoneum (5.4%) and kidney (2.7%) (*n*=40). We confined our subsequent analysis to the liver as it is the most frequent site of metastasis. Lesions were binned into five size categories according to the number of YFP^+^ cells: single, nano (2–10 cells), micro (11–100 cells), milli (101–1000 cells) and macro (greater than 1000 cells) (Fig. 1a). When examined microscopically, nearly all tumour-bearing KPCY mice exhibited single cells (95.6%) or nano-metastases (91.3%) in the liver, while a lower frequency of animals had micro-metastases (65.2%) or milli- and/or macro-metastases (39.1%) (*n*=23). Most metastatic lesions contained 100 or fewer cells, with nano- and micro-metastases being the most abundant (Fig. 1b). Detailed metastatic burden quantification for each animal is listed in Supplementary Table 1.

We next sought to determine baseline levels of proliferation and apoptosis within metastatic lesions. Surprisingly, proliferation rates (as measured by the percentage of Ki67^+^ cells within each lesion) were not significantly different across lesions of all five metastatic categories (11–19%) and were comparable to the primary tumour (Fig. 1c, Supplementary Fig. 1a). These results were corroborated by staining for phospho-histone H3 (pH3), a marker of mitosis (Supplementary Fig. 1b,e) and proliferating cell nuclear antigen (Supplementary Fig. 1c,f). Apoptosis, as measured by the percentage of cleaved caspase-3 (CC3) positive cells within each lesion, was absent from single cells and rarely seen in nano-metastases (0.2% of cells within nano-metastases were CC3^+^). Primary tumours and larger lesions also exhibited low (∼1%), albeit significantly higher levels of apoptosis compared to single cells (Fig. 1c; Supplementary Fig. 1d). To determine if proliferation is also constant across lesions in human PDAC we stained matched primary tumours, gross metastases and micro-metastatic lesions from 7 patients for Ki67 (Supplementary Fig. 2a–d). We found that the rate of Ki67 positivity (mean±s.d.) was not significantly different between primary tumours (2.1±1.9), gross metastases (5.4±5.6) and micro-metastases (3.1±5.9), similar to what we observed in mouse PDAC. These data suggest that metastatic cancer cells exhibit similar rates of proliferation regardless of lesion size.

### Metastatic lesions become more epithelial as they grow

**Figure 2:**
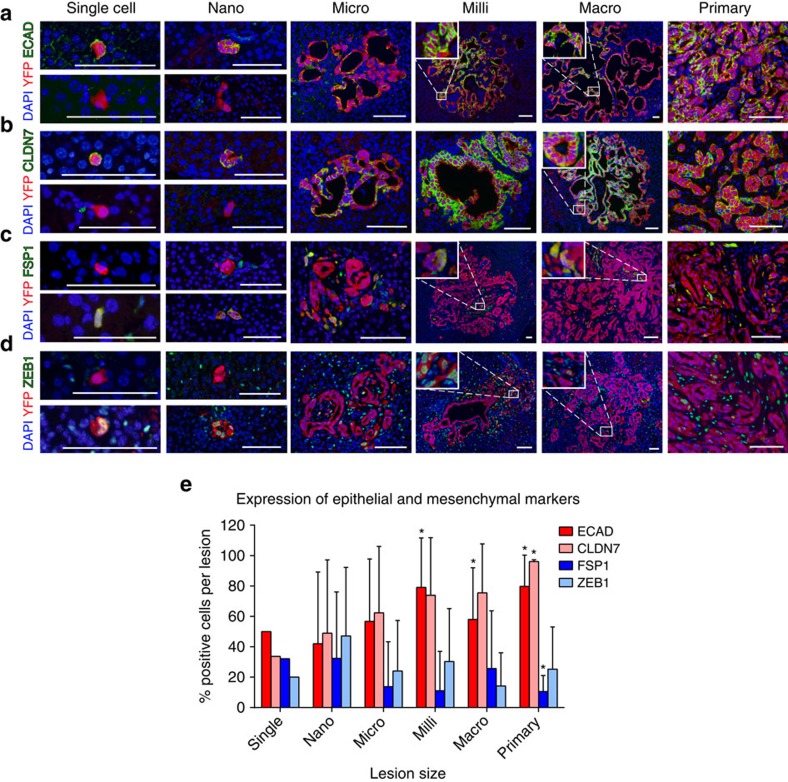
Metastatic growth is associated with a more epithelial phenotype. (**a**–**d**) Representative images of metastases and primary tumour stained for DAPI (blue), YFP (red) and ECAD (**a**), CLDN7 (**b**), FSP1 (**c**) and ZEB1 (**d**) (green). Scale bars, 50 μm. (**e**) Quantification of EMT in metastases. The percent of positive cells for each lesion was determined and the percentages were averaged across lesions of the same size (ECAD, *P*<0.05; CLDN7, NS; FSP1, *P*<0.05; ZEB1, NS). *n*≥8 mice, ≥100 lesions for each stain. Data are presented as mean±s.d. *P*-values were calculated by one-way ANOVA and one sample *t*-tests against ‘single cell' means; *P<0.05.

We next determined the epithelial-mesenchymal status of cells at various stages of metastatic growth by costaining with a panel of epithelial and mesenchymal markers and the YFP lineage label. To identify tumour cells with epithelial characteristics, we used antibodies that recognize E-cadherin (ECAD) and Claudin-7 (CLDN7)^18^,^19^,^20^ and measured the percentage of YFP^+^ cells that exhibited positive staining as a function of lesion size. Compared to single cells and nano-metastases, in which fewer than half of the cells were positive for these markers, metastases having 100 cells or more (milli- and macro-) exhibited rates of epithelial staining in the range of 60–80%, resembling primary tumours (Fig. 2a,b,e). A complementary pattern of staining was observed when antibodies against fibroblast-specific protein-1 (FSP1) or zinc finger E-box binding homeobox 1 (ZEB1) were used^21^,^22^, as milli- and macro-metastases exhibited reduced staining with these markers relative to nano-metastases (Fig. 2c–e). Comparing the frequency of epithelial (ECAD/CLDN7) cells to mesenchymal (FSP1/ZEB1) staining within each size group revealed that single cells and nano-metastases exhibited comparable frequencies of cells positive for these epithelial and mesenchymal markers; by contrast, larger lesions had a significantly greater fraction of epithelial cells (Supplementary Fig. 3). These results are consistent with the notion that small metastatic lesions contain a higher percentage of cells that have undergone EMT, and that such cells revert to a more epithelial state, via MET, during colonization and growth. To determine whether this trend is also observed in human PDAC, we performed IHC on primary human PDAC tumours, gross liver metastases and microscopic liver metastases for CLDN7 and FSP1 (*n*=6 cases). Consistent with the murine data, CLDN7 staining was greater in primary tumours than micro-metastases while FSP1 staining was greater in micro-metastases compared to gross metastases and primary tumours (Supplementary Fig. 4). Thus, in both mouse and human PDAC, metastatic cells appear to re-acquire an epithelial phenotype with increasing lesion size.

### Desmoplasia gradually accumulates at metastatic lesions

**Figure 3:**
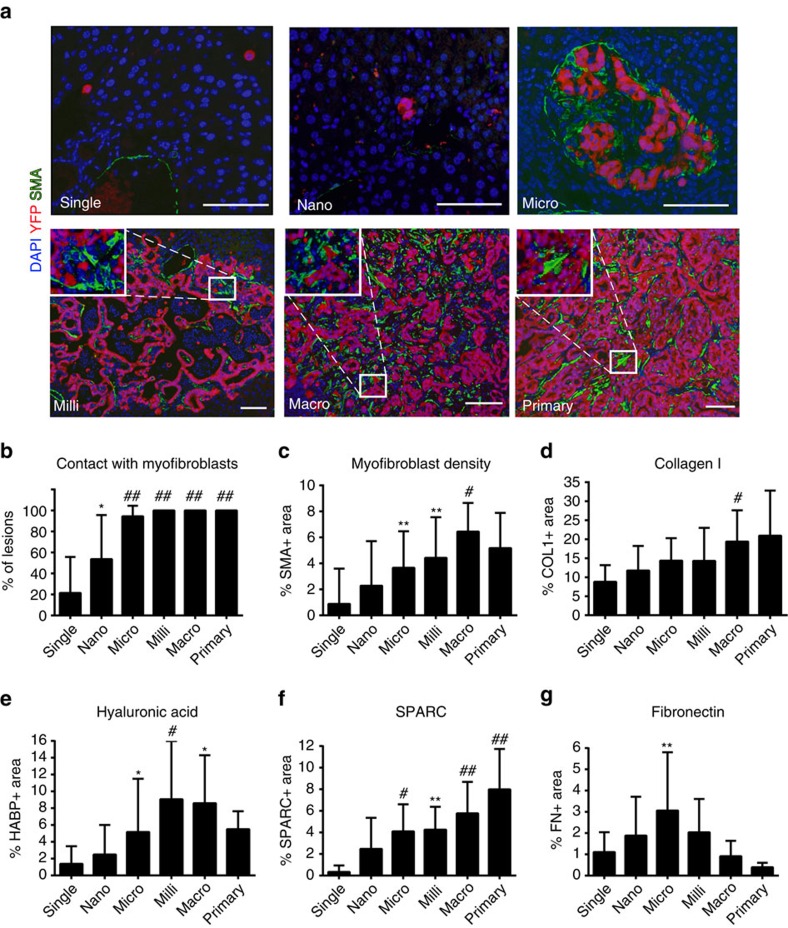
Desmoplasia accumulates as lesions grow. (**a**) Representative images of metastases and primary tumour stained for DAPI (blue), YFP (red) and α-SMA (green). Scale bars, 50 μm. (**b**) Contact between metastases and α-SMA^+^ fibroblasts. Each lesion was binned by size and scored for direct contact with an α-SMA^+^ cell (*n*=9 mice, 167 lesions). (**c**) α-SMA^+^ fibroblast density at metastatic lesions. Percent α-SMA^+^ area was quantified within one cell diameter of metastatic lesions (*n*=9 mice, 167 lesions). (**d**–**g**) Extracellular matrix (ECM) density at metastatic lesions. Metastatic livers were stained for ECM components COL1 (**d**), HABP (**e**), SPARC (**f**) and FN (**g**). Percent positive area was quantified within one cell diameter of each lesion. *n*≥5 mice, ≥50 lesions for each stain. Data are presented as mean±s.d. *P*-values were calculated by one-way ANOVA and one sample *t*-tests against ‘single cell' means; *P<0.05; **P<0.01; ^#^*P*<0.001; ^##^*P*<0.0001.

Desmoplasia—a dense infiltrate of non-cancerous stromal cells and ECM components—is a hallmark of PDAC, where it is thought to promote tumour growth and act as a barrier to the effective delivery of chemotherapy^23^. Myofibroblasts, a subset of fibroblasts involved in wound healing and fibrosis, are among the most prevalent stromal cell types in PDAC. To determine if and when myofibroblasts accumulate during metastatic growth, we stained metastatic livers for YFP and α-smooth muscle actin (αSMA). Importantly, and in contrast to a recent publication^24^, αSMA is specific to myofibroblasts and is never expressed in cancer cells that have undergone EMT (under review). Nearly all metastatic lesions consisting of 10 or more cells were in direct contact with myofibroblasts, while smaller lesions were less frequently associated, especially single cells (Fig. 3a,b). In particular, myofibroblast recruitment seemed to occur at the nano-metastasis stage, as there was a direct correlation between the number of cells present in a lesion (from 2 to 10) and their association with myofibroblasts (Supplementary Fig. 5a). Additionally, the number of associated myofibroblasts significantly increased with lesion size, as demonstrated by an increase in αSMA^+^ area at larger lesions (Fig. 3c).

ECM components can be deposited by myofibroblasts or by tumour cells themselves (following EMT), and contribute to the desmoplastic reaction in PDAC^14^,^25^. Collagen I (COL1), hyaluronic acid (HA), fibronectin (FN) and secreted protein acidic and rich in cysteine (SPARC) represent the most abundant constituents in PDAC^25^,^26^. With the exception of FN density, which peaked at intermediate-sized micro-metastases, all other ECM components exhibited an increase as lesions grew (Fig. 3d-g, Supplementary Fig. 5b-e) with the most dramatic change being the levels of SPARC present in all lesions as compared to single cells (Fig. 3f). These results demonstrate that the desmoplastic response begins at the nano-metastasis stage and that most stromal components increase in density as a function of lesion size. We also sought to determine whether ECM accumulation occurs during metastatic growth in human PDAC. We performed IHC for FN on primary human PDAC tumours, gross liver metastases and microscopic liver metastases and found that FN expression is higher in micro-metastases compared to primary tumours (Supplementary Fig. 6), consistent with the pattern of FN deposition observed in KPCY metastases.

In addition to myofibroblasts and ECM, pancreatic neoplasia is accompanied by a robust infiltration of leukocytes, particularly myeloid cells, which can comprise more than half of the cells within a tumour^27^,^28^. We thus determined how leukocyte populations change during metastatic progression by staining livers for CD45 (pan-leukocyte), F4/80 (macrophages), Gr-1 (myeloid-derived suppressor cells (MDSCs)), CD3 (T lymphocytes) and YFP (Supplementary Fig. 7). Although the number of macrophages, MDSCs and leukocytes in general was increased in uninvolved areas of metastatic livers compared to control (Pdx1-Cre; Rosa^YFP^) livers (Supplementary Fig. 7a-e), only MDSCs showed a significant association with metastatic lesions, specifically nano-, micro- and milli-metastases (Supplementary Fig. 7h,m). In contrast, T lymphocyte density, which was lower overall compared to the other leukocyte subsets, was unchanged between metastatic and control livers and across metastatic lesions (Supplementary Fig. 7j,n). These results suggest that the presence of a primary pancreatic tumour causes a marked increase in certain leukocyte subsets in the liver, only MDSCs exhibit dynamic changes during metastatic progression.

### Metastatic growth is associated with hypovascularity

**Figure 4:**
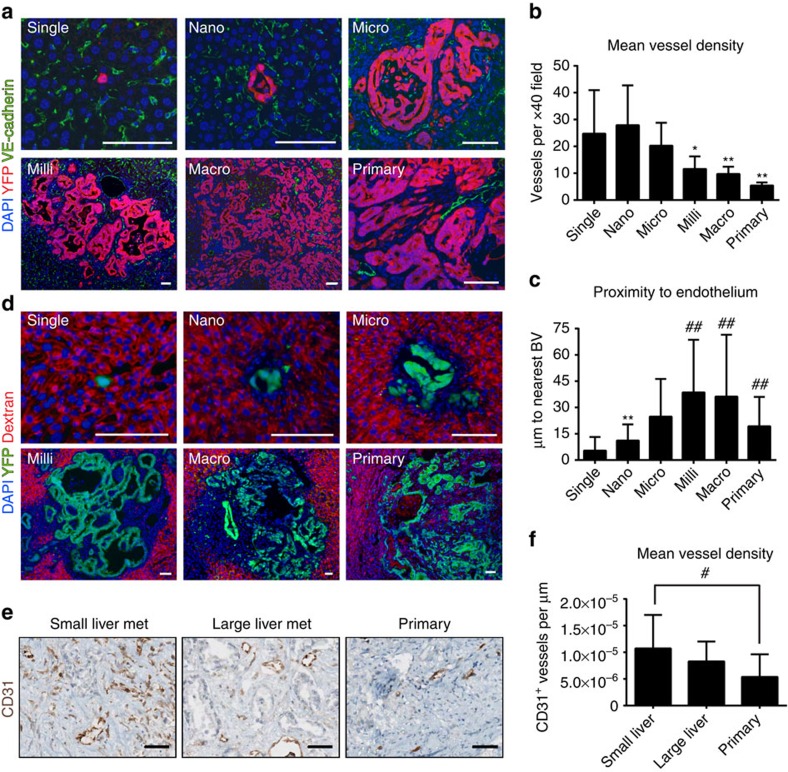
Metastatic growth is associated with functional hypovascularity. (**a**) Representative images of metastases and primary tumour stained for VECAD (green), YFP (red) and DAPI (blue). Scale bars, 50 μm. (**b**) Metastatic vessel density. VECAD^+^ blood vessels were quantified at metastases within × 40 fields. Data are presented as mean±s.d.; *n* =7 mice, 100 lesions. (**c**) Proximity to blood vessels. For each lesion, the distance between five random tumour cells and the nearest VECAD^+^ blood vessel was determined. Data are presented as mean±s.d. *n*=7 mice, 100 lesions. *P*-values were calculated by one-way ANOVA and one sample *t*-tests against ‘single cell' means. (**d**) Functional hypovascularity at large metastases. Representative images of fluorescent dextran accumulation (red) at metastatic lesions (YFP, green; DAPI, blue). (**e**) Representative images of human primary PDAC and matched liver metastases stained for CD31 (brown). Scale bars, 50 μm. (**f**) Mean vessel density quantification of human primary PDAC and matched liver metastases (*n*=25 cases). Data are presented as mean±s.d.; *P<0.05; **P<0.01; ^#^*P*<0.001; ^##^*P*<0.0001.

PDAC tumours are commonly hypovascular, leading to increased hypoxia as well as impaired drug delivery^29^,^30^. To characterize the vascular properties of metastatic lesions, we stained metastatic livers for VE-cadherin (VECAD) and calculated vessel density (VECAD^+^ lumen-containing blood vessels per × 40 field). As expected given the high prevalence of vascular sinusoids in the normal liver, vessel density in the vicinity of small lesions (single cells and nano-metastases) was high, ranging between 25 and 30 vessels per field (Fig. 4a,b). In bigger lesions, however, vessel density decreased, approaching the mean of five vessels per field found in primary tumours (Fig. 4a,b). Quantification of the average distance (mean±s.d.) from metastatic cells to the closest VECAD^+^ vessel produced a similar trend: single cells (*n*=34) and nano-metastases (*n*=130 cells) were in close proximity to blood vessels (mean distance 5.4±7.7 and 11.1±9.2 microns, respectively) while cells within milli- (*n*=115 cells) and macro-metastases (*n*=60 cells) were far removed from the nearest blood vessel (mean distance 38.5±30.1 and 36.2±35.3 microns, respectively) similar to primary tumours (mean distance 19.2±16.7 microns, *n*=213 cells) (Fig. 4c).

To assess whether these observations result in functional differences in perfusion, we injected tumour-bearing KPCY mice with Texas-red dextran, a high molecular weight fluorescent polysaccharide. Consistent with the observation that metastatic lesions become more hypovascular as they grow, we observed a ‘halo' of dextran-poor areas that became more prominent with increased size (Fig. 4d). These data demonstrate that metastatic PDAC liver lesions are initially well-vascularized and perfused but become progressively hypovascular with growth. To determine if this trend of decreasing vascularity with increasing lesion size holds true in human disease, we stained human PDAC tumours and matched small and large liver metastases for CD31 and calculated vascular density as CD31^+^ vessels per micron. Consistent with our findings in the KPCY model, small but not large metastases had a higher vessel density compared to primary tumours (Fig. 4e,f). Thus, in both the KPCY model and human PDAC, metastatic growth is associated with decreasing vascular density.

### The impact of chemotherapy on metastasis

**Figure 5:**
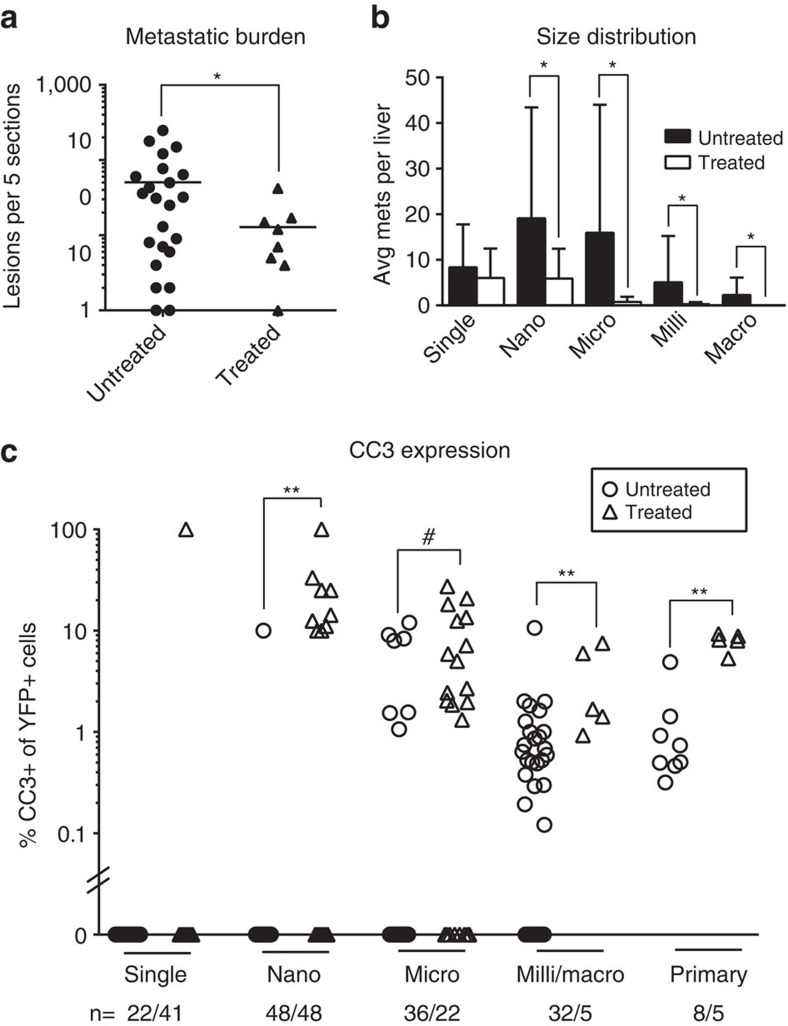
Chemotherapy reduces metastatic burden. (**a**) Liver metastatic burden after long-term chemotherapy. Each dot represents one mouse. Untreated group consists of historical controls from Fig. 1b (untreated, *n*=23; treated, *n*=8). Bars represent means±s.d.; *P<0.05, Student's *t*-test with Welch's correction. (**b**) Size distribution of metastatic lesions after long-term chemotherapy. Data are presented as mean±s.d., *P<0.05, Student's *t*-test with Welch's correction. (**c**) Apoptosis rates in metastases after single dose chemotherapy. CC3 positivity in metastases was assessed 10-12 h after treatment. Each dot represents a lesion; line represents the mean. *P<0.05 by Mann-Whitney test.

The studies described so far reveal that large metastatic lesions are hypovascular and surrounded by dense, desmoplastic stroma while small lesions and single cells, although well-perfused by nearby liver sinusoids, have not yet established a local tumour microenvironment. As PDAC stroma has been proposed to act as a physical barrier to drug delivery in primary tumours^29^,^30^, we hypothesized that large metastases would be more resistant to chemotherapy compared to small lesions and single cells. To test the ‘stromal barrier' hypothesis, we treated tumour-bearing KPCY mice with standard of care chemotherapy—the nucleoside analog gemcitabine (GEM) and the albumin-bound microtubule inhibitor nab-paclitaxel (PTX)—to examine the effects of chemotherapy on metastasis. Animals received intraperitoneal injections of GEM and PTX (each at 120 mg kg^−1^) every four days for 2–4 weeks, for a total of 4–8 doses of chemotherapy, and were sacrificed when moribund. Compared to untreated historical controls (matched for age and tumour weight; Supplementary Fig. 8a,b), this treatment regimen led to a dramatic reduction in metastatic tumour burden with a decrease in the mean (±s.d.) number of lesions from 50.7±64.9 (untreated) to 13.3±13.0 (treated) (Fig. 5a). In addition, the metastases that did form were significantly smaller (Fig. 5b), with no macro-metastases and only a single milli-metastasis evident in chemotherapy-treated animals. Interestingly, chemotherapy also led to a reduction in the frequency of circulating tumour cells (CTCs) in tumour-bearing KPCY mice, with a log decrease in the mean number of CTCs from 141.6±390.0 per ml (untreated) to 14.6±14.6 per ml (treated) (Supplementary Fig. 8c).

We reasoned that GEM/PTX could shift the numerical and size distribution of metastases by either preventing the progression of small lesions or by reducing the burden of multicellular metastatic lesions at every stage. To distinguish between these possibilities, we treated tumour-bearing KPCY mice with a single dose of chemotherapy and assessed cell death in primary tumours and metastases 10–12 h later by CC3 staining. With the exception of single cells, metastases in all size categories exhibited a significant increase in CC3 staining after treatment (Fig. 5c, Supplementary Fig. 8d). Surprisingly, almost no cell death was observed in single YFP^+^ cells following GEM/PTX treatment (Fig. 5c), consistent with the observation that the number of single cell lesions did not change after chemotherapy (Fig. 5b). These data suggest that chemotherapy induces comparable degrees of cell death in lesions of all sizes greater than one cell.

We considered the possibility that EMT is responsible for the apparent chemoresistance of single cells. EMT has been implicated in resistance to GEM in PDAC^31^,^32^,^33^, and since small metastases have high rates of EMT (Fig. 2), we hypothesized that lesions treated with GEM/PTX for 2–4 weeks would be enriched for mesenchymal features. We assessed the expression of ECAD and FSP1 in untreated and treated primary tumours and small metastatic lesions, focusing only on single cells and nano-metastases since nearly all treated lesions fell into these two size categories (representative images in Supplementary Fig. 9a,c). Surprisingly, treated primary tumours and metastatic lesions were significantly depleted of ECAD^−^ and FSP1^+^ mesenchymal tumour cells (Supplementary Fig. 9b,d). We then assessed the rate of apoptosis in ECAD^+^ and ECAD^−^ tumour cells from mice treated with one dose of GEM/PTX and harvested 10–12 h later. Consistent with the loss of mesenchymal tumour cells with long-term treatment, ECAD^−^ tumour cells in the primary tumour and metastases had significantly higher rates of CC3 positivity compared to ECAD^+^ cells (Supplementary Fig. 9e,f). These data suggest that EMT promotes chemosensitivity in the context of GEM/PTX treatment.

## Discussion

The highly inefficient nature of metastasis has made it difficult to observe the cellular and molecular events underlying tumour cell spread and growth at distant sites. As a result, most animal studies of metastasis have relied on transplantation of tumour cell lines into recipient animals. In this study, we used a genetically engineered mouse model to characterize the key events that accompany metastatic growth, from single cells to large grossly detectable metastases. Importantly, the stochastic nature of tumour development and metastatic progression in the KPCY model allowed us to assess the natural history, and treatment response, of metastasis in the setting of naturally evolving and genetically heterogeneous tumours, an approach that has not been taken previously with any cancer type.

The vast majority of animals with advanced pancreatic tumours had either overt or occult metastases, mirroring the human disease in which relapse is the norm despite surgery, with most lesions being smaller than 100 cells in size. Although small metastatic lesions exhibited features that distinguished them from primary tumours, they progressively acquired cell-intrinsic and -extrinsic features of the primary tumour. One of the biggest surprises of our study was that the rate of cell proliferation did not fluctuate with lesion size despite the dramatic changes in the microenvironment with progression. Large lesions were predominantly comprised of tumour cells with an epithelial phenotype, an observation consistent with the notion that epithelial properties are advantageous at metastatic sites^11^,^12^,^13^. Because proliferation rates did not vary across lesions and death rates were negligible, the tendency for large metastatic lesions to have an epithelial phenotype is most likely the result of MET rather than selective outgrowth of the epithelial population. Although the factors that drive EMT and MET *in vivo* remain to be determined, the observation that lesions acquire stroma as they grow represents one potential source of signals.

Myofibroblasts appear early during metastatic growth, with most lesions having direct contact with myofibroblasts by the time they are 6–7 cells in size. This rapid recruitment suggests that factor(s) produced by the cancer cells either attract pre-existing myofibroblasts to the lesion or activate local stellate cells to differentiate into myofibroblasts. ECM deposition became more evident in advanced lesions, with macro-metastases exhibiting levels of collagen I, fibronectin, hyaluronic acid and SPARC comparable to levels present in primary tumours, consistent with a recent study reporting similar levels of myofibroblasts, collagen and hyaluronic acid in human primary pancreatic cancers and large metastases^34^.

The vasculature also underwent dynamic changes during metastatic progression, with small lesions surrounded by blood vessels and larger lesions exhibiting hypovascularity, mirroring the hypovascular nature of primary PDACs in this model and in the majority of patients. Importantly, we found that human pancreatic tumours exhibit the same phenomenon, with small metastatic lesions having a higher vessel density than large lesions and primary tumours. Although the mechanism leading to vessel paucity in primary tumours and large metastases remains unknown, we recently showed that depletion of stromal fibroblasts (by interfering with Shh signalling) results in increased vessel density, suggesting that fibroblasts and/or ECM components exert an anti-angiogenic effect^35^. Hence, it is possible that hypovascularity is a consequence of increased myofibroblast activity. Regardless of the mechanism, it appears that the so-called ‘angiogenic switch'—whereby tumour cells activate an angiogenic signal to increase their vascularity—is dispensable for the transition from micro-to macro-metastasis in PDAC.

For patients with metastatic PDAC, treatment with chemotherapy provides minimal improvement of survival^36^,^37^, whereas adjuvant chemotherapy for patients in clinical remission after initial resection offers a more substantial benefit, doubling overall survival. In the KPCY model, we observed killing in a wide spectrum of metastases, providing direct evidence that adjuvant chemotherapy targets lesions that are too small to detect by standard imaging. We had initially hypothesized that small lesions, with their close proximity to endothelium and lack of stroma, would be particularly susceptible to chemotherapy, while large lesions, which are hypovascular and protected by stroma, would be resistant^29^,^30^,^38^. Thus, it was surprising that chemotherapy resulted in comparable levels of cell death in small lesions, large lesions and primary tumours, while having only minimal impact on single cells. These results suggest that the stromal barrier hypothesis—which postulates that blood vessel paucity and desmoplastic stroma impedes the delivery of chemotherapy—is incomplete, at least with respect to metastatic lesions.

There are several possible explanations for the apparent resistance of single cells to both long-term and short-term chemotherapy. The simplest possibility is that single disseminated cells are replenished by the primary tumour faster than they are being removed by chemotherapy. However, the observation that treated mice have significantly fewer CTCs would argue against this hypothesis. Mesenchymal tumour cells were acutely sensitive to chemotherapy, consistent with the observation by Collisson *et al.* that human and murine mesenchymal PDAC cell lines are more susceptible to gemcitabine compared to epithelial lines^39^. Single cells were enriched for epithelial features after long-term treatment, and since the average number of single cells did not differ between untreated and treated mice this likely reflects an MET. Therefore, another possibility is that the epithelial plasticity of single disseminated cells plays a role in resistance to the combined treatment of gemcitabine and nab-paclitaxel. Finally, as nab-paclitaxel is thought to act by binding SPARC in the tumour microenvironment, increasing its local concentration, it is possible that the absence of SPARC near single cells spares them from cytotoxicity. This is an especially attractive mechanism in light of the fact that EMT induces SPARC expression^40^, and there is significantly less EMT within treated lesions. While SPARC's role in primary tumour chemosensitivity is disputed^41^,^42^,^43^,^44^, its role in metastasis is yet to be explored.

Our data also have implications for the treatment of patients with resectable PDAC. KPCY animals treated for only 2–4 weeks with gemcitabine/nab-paclitaxel exhibited a marked improvement in metastatic burden, yet no animals had complete absence of metastatic disease. Indeed, this failure of combination chemotherapy to demonstrate ‘curative potential' in KPCY mice mirrors a critical failing of adjuvant chemotherapy for patients with PDAC; namely, improvement in median survival compared to observation, yet only rare long-term remissions or cures. Thus, even as gemcitabine/nab-paclitaxel and other combinations are beginning to be tested in the adjuvant clinical setting, our findings provide additional motivation to evaluate novel, non-chemotherapeutic approaches that target residual micrometastatic disease.

## Methods

### Mouse strains

Pdx1-cre, Kras^LSL−G12D^, p53^L/+^, Rosa^YFP/YFP^ mice have been described previously^15^ and were bred and maintained at the University of Pennslyvania Small Animal Facility. Beginning at the age of 12 weeks, KPCY mice were palpated and examined for evidence of morbidity twice per week. Tumour-bearing animals were sacrificed when moribund. Both male and female mice were used for analysis with a mean age of 22.6±8.1 weeks. Pdx1-cre, Rosa^YFP/YFP^ mice were used as controls. All vertebrate animal experiments were conducted in compliance with the National Institutes of Health guidelines for animal research and approved by the University of Pennsylvania Institutional Animal Care and Use Committee.

### Immunofluorescence

Tissues were fixed in Zn-formalin, paraffin embedded and stained as previously described^15^. In brief, after sections were deparaffinized, rehydrated and subjected to antigen retrieval, sections were blocked in 5% donkey serum for 1 h at room temperature (RT), incubated with primary antibodies for 1 h at RT, washed, incubated with secondary antibodies for 1 h at RT, washed and mounted. Rabbit anti-Zeb1 (Santa Cruz Biotechnology, Santa Cruz, CA) required additional tyramide signalling amplification (PerkinElmer, Waltham, MA). See Supplementary Table 2 for a list of antibodies used. Slides were visualized using an Olympus IX71 inverted multicolour fluorescent microscope.

### Metastasis quantification

Gross metastases were confirmed by fluorescent microscopy using a Leica MZ16FA multi-colour fluorescent stereomicroscope. To determine metastatic burden, YFP^+^ lesions were quantified for five liver sections spaced 100 μm apart. Large metastatic lesions that were captured on multiple sections were only counted once.

### Percent area quantification

Percent area was determined for a subset of stains (αSMA, COL1, HA, SPARC, FN, CD45, F4/80, GR-1, CD3 and VECAD) by first cropping the image within one cell diameter of each metastatic lesion. Using ImageJ, fluorescent channels were split and the channel of interest was thresholded to highlight positive staining while excluding background. The ‘analyse particles' tool was then used to calculate percent area.

### Fluorescent dextran administration

Female tumour-bearing mice between 12–14 weeks of age were injected with 25 μg kg^−1^ Texas red-conjugated dextran (70,000 MW; Thermofisher Scientific, Waltham, MA) 30 min prior to euthanasia. Tissues were embedded in OCT (Electron Microscopy Sciences, Hatfield, PA), cut into 5 μm sections and imaged on an Olympus IX71 inverted multicolour fluorescent microscope.

### Human specimens

Tissue samples were obtained from patients who consented to a research autopsy in association with the IRB approved Johns Hopkins Rapid Medical Donation Program (PMID: 19273710). Clinicopathological characteristics are listed in Supplementary Table 3. Formalin-fixed and paraffin-embedded samples of the primary carcinoma, two independent gross liver metastasis sections from these patients were used for image analysis and immunohistochemistry. Micrometastases were detected within the normal liver parenchyma adjacent to gross liver metastases and were identified by nuclear atypia and larger cell size.

### Immunohistochemistry

Sections were deparaffinized in xylene, rehydrated and subjected to antigen retrieval. Endogenous peroxidases were blocked with 1.5% H_2_O_2_. Endogenous avidin and biotin were also blocked using an Avidin/Biotin Blocking Kit (Vector Labs, Burlingame, CA) according to the manufacturer's instructions. Sections were then blocked with 5% donkey serum in 0.3% Triton-X100 (MP Biomedicals, Santa Ana, CA) in PBS for 1 h at RT, then incubated with primary antibodies at 4 °C overnight. The next day, slides were washed in 0.1% Tween-20 (Fisher Scientific, Pittsburgh, PA) in PBS (PBST) and incubated with a biotin-conjugated secondary antibody for 1 h at RT. Slides were washed in PBST and staining was revealed using ABC-HRP and DAB kits (Vector Labs) according to the manufacturer's instructions.

### Human CD31 quantification

Immunolabelled slides were scanned at × 200 total magnification at a resolution of 0.49 μm per pixel using an Aperio Scanscope CS digital slide scanner (Aperio Technologies, Inc., Vista, CA). Aperio ImageScope was used to extract 735 μm^2^ (1500 × 1500 pixel) fields as TIFF images for further analysis. The fields were randomly selected in the area of interest using a low magnification view of the slide. To compare vessel density in primary and metastatic tumour, up to five fields were extracted from each slide. The extracted fields were then analysed with image analysis software written using ImageJ (Wayne Rasband, NIH, http://rsbweb.nih.gov/ij/). Total tissue area was measured by manually thresholding a grayscale version of the original field. The CD31-positive area was then measured by first masking out any areas with background staining using manual drawing tools and then performing colour deconvolution to separate the DAB and hematoxylin staining^45^. The DAB image was then manually thresholded to select the CD31 positive pixels. Particles (groups of connected pixels) less than 150 pixels (73.5 μm^2^) in size were excluded to reduce the degree of large vessel fragmentation and the presence of single immunoreactive cells. CD31^+^ vessel density was calculated as the number of CD31 positive particles/total tissue area.

### Chemotherapy administration

Tumour-bearing animals in healthy condition were enrolled once the tumour had reached an estimated × 1 cm in diameter based on palpation. With the knowledge that the average number of metastases per mouse in our historical, untreated controls is 50.7 (±64.9), we concluded that a large effect size of at least a 75% decrease in metastasis with a relatively small (≤10) standard deviation would be necessary to detect significant changes after treatment. Using these values, with an alpha error level of ≤5%, we determined that a sample size of eight mice would be sufficient. Gemcitabine-HCl (Sun Pharmaceuticals, Mumbai, India) and nab-paclitaxel (Abraxane; Celgene, Summit, NJ) were both dissolved in PBS and administered via intraperitoneal injection at 120 mg kg^−1^. The drugs were administered simultaneously once every four days until the mouse became moribund, between 2 and 4 weeks from enrolment. Each mouse received at least 4 (and a maximum of 8) doses.

### Flow cytometry

Blood was collected by cardiac puncture as previously described^15^. Erythrocytes were removed using RBC lysis buffer (G-biosciences, St Louis, MO) according to the manufacturer's instructions. The remaining cellular fraction was stained with APC anti-mouse CD45 (Biolegend, San Diego, CA) and analysed on a BD FACSVerse flow cytometer. DAPI^−^/CD45^−^/YFP^+^ events were counted as CTCs.

### Statistical analysis

Differences between two groups were analysed by two-tailed Student's t-test with Welch's correction to account for unequal SDs, or by Mann-Whitney test for non-normally distributed data. Differences between three or more groups were analysed by one-way ANOVA with Tukey's multiple comparisons test used as a *post hoc* test to assess differences between ‘single cells' and all other groups. For experiments in which ‘single cell' means were binary (0 or 100%), differences between ‘single cells' and all other groups were analysed by one-sample *t*-test. All statistical analyses were performed using GraphPad Prism 6 (GraphPad, La Jolla, CA). *P*≤0.05 denotes differences that are statistically significant.

### Data availability

The data that support the findings of this study are available within the Article and Supplementary files or available from the corresponding authors on request.

## Additional information

**How to cite this article**: Aiello N. M. *et al.* Metastatic progression is associated with dynamic changes in the local microenvironment. *Nat. Commun.* 7:12819 doi: 10.1038/ncomms12819 (2016).

## Supplementary Material

Supplementary Information Supplementary Figures 1-9, Supplementary Tables 1-3
