# Supplementary material for: Metastatic progression is associated with dynamic changes in the local microenvironment
Source: Nat Commun. 2016 Sep 15;7:12819. doi: 10.1038/ncomms12819 (PMC5027614; doi:10.1038/ncomms12819)
Supplement: Supplementary Information — Supplementary Figures 1-9, Supplementary Tables 1-3 [file ncomms12819-s1.pdf]

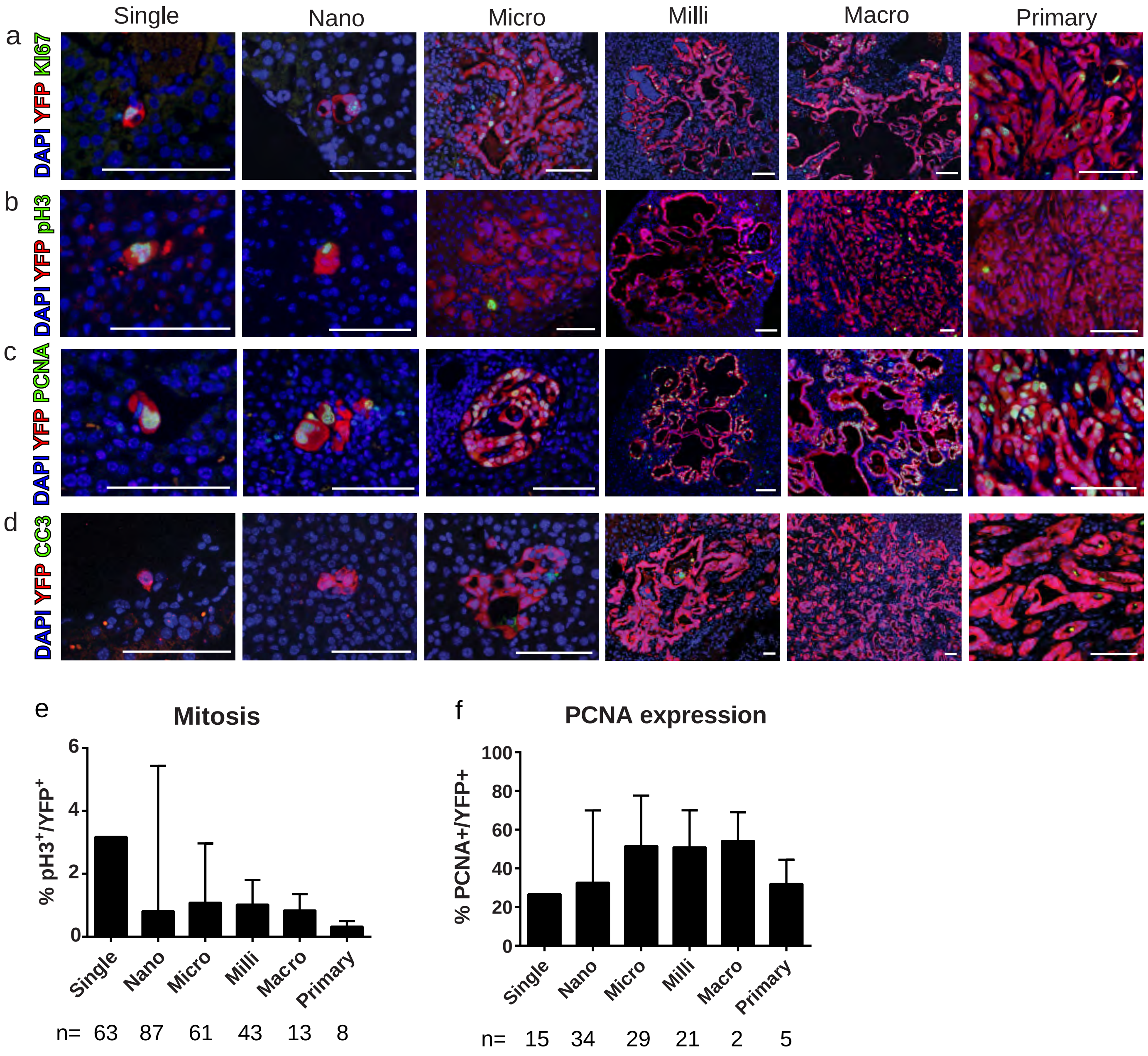

Supplementary Figure 1. Proliferation/apoptosis rates of metastatic lesions. (**a**) Proliferation. Primary tumor and metastases were stained for YFP (red), DAPI (blue) and Ki67 (green). (**b**) Mitosis. Primary tumor and metastases were stained for YFP (red), DAPI (blue) and pH3 (green). (**c**) PCNA expression. Primary tumor and metastases were stained for YFP (red), DAPI (blue) and PCNA (green). (**d**) Apoptosis. Primary tumor and metastases were stained for YFP (red), DAPI (blue) and CC3 (green). (**e**) Quantification of pH3 staining; p= 0.9633. (**f**) Quantification of PCNA staining; p=0.0642. Bars represent means ± SD. Scale bars, 50 μm.

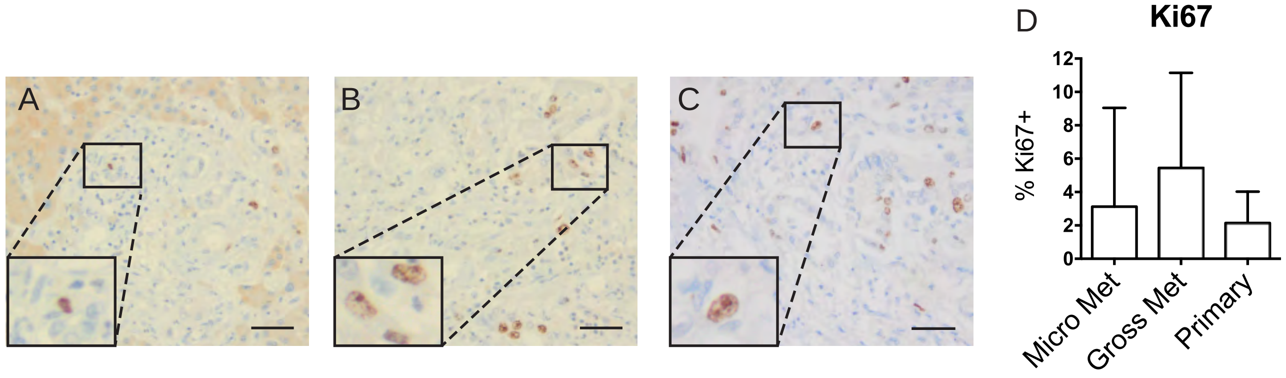

Supplementary Figure 2. Proliferation in human PDAC tumors and metastases. (**a-c**) Representative images of a micrometastatic lesion (a), gross metastasis (b) and primary tumor (c) stained for Ki67 by IHC. (d) Quantification of Ki67 staining. The percentage of Ki67$^+$ cells was determined for micro-metastases (n=126), gross metastases (n=11) and primary tumors (n=7); p=0.389. For large lesions (gross metastases and primary tumors), five fields were averaged together. Bars represent means ± SD; **, p<0.01.

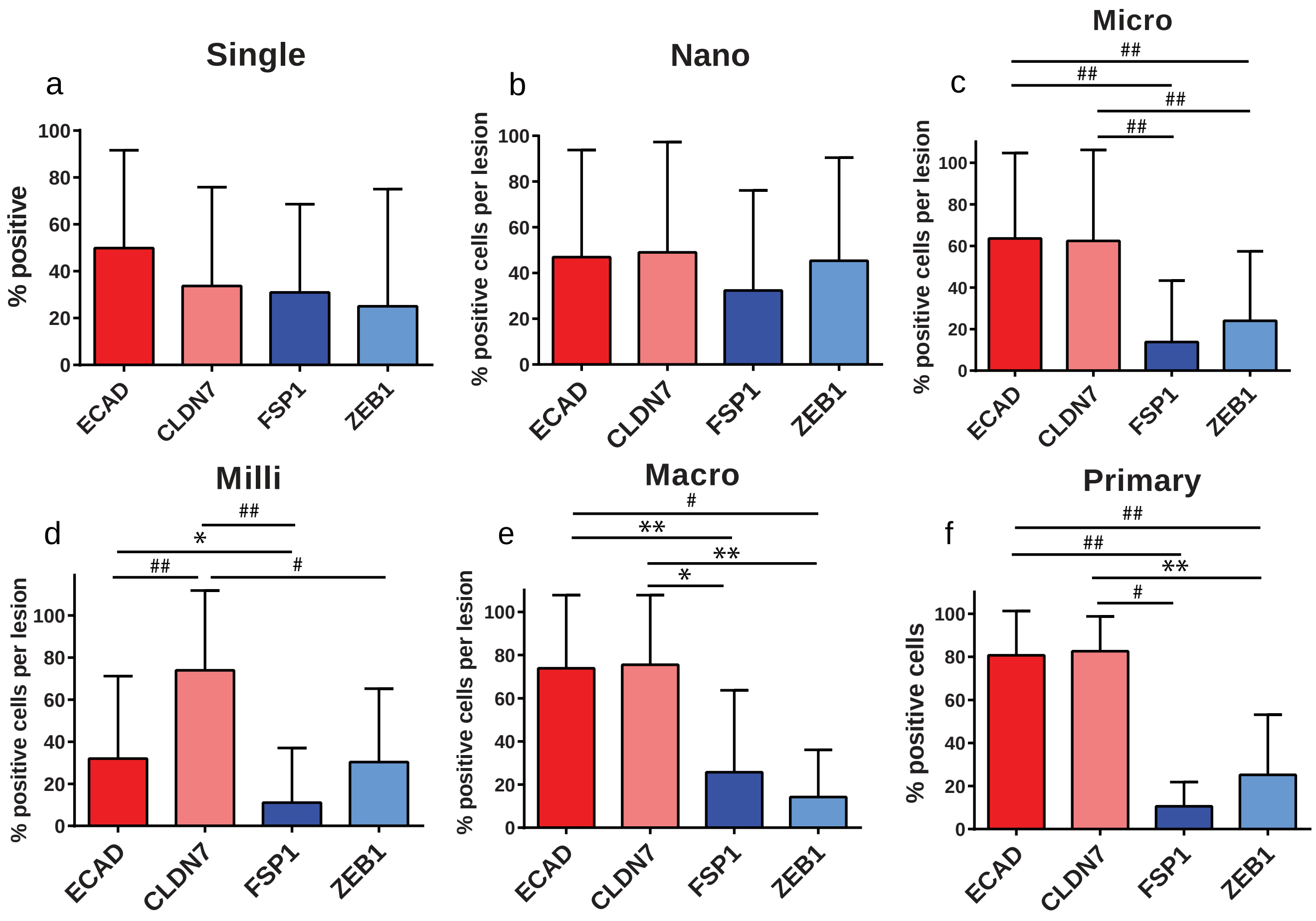

Supplementary Figure 3. Skewing of epithelial to mesenchymal cell ratio within primary tumor and metastatic lesions. (**a-f**) Comparison of epithelial (ECAD+, CLDN7+) and mesenchymal (FSP1+, ZEB1+) cell frequency within each size category. Bars represent means ± SD; *, p<0.05; **, p<0.01; #, p<0.001; ##, p<0.0001.

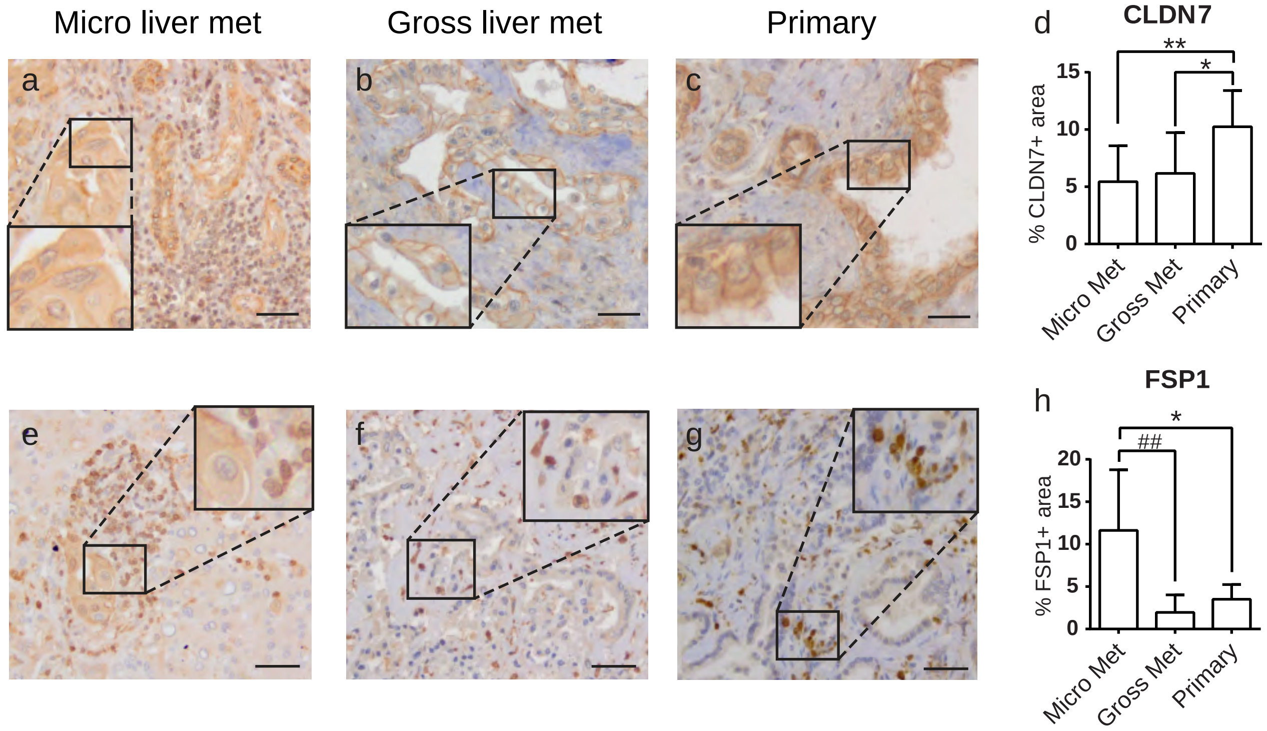

Supplementary Figure 4. Epithelial and mesenchymal features of human PDAC tumors and metastases. (**a-c**) Representative images of human microscopic liver metastasis (n=114), gross liver metastasis (n=24) and primary PDAC tumors (n=6) stained for CLDN7 by IHC. (**d**) Quantification of CLDN7+ area per 20X field for gross metastases and primary tumors or within one cell diameter for micro-metastases. (**e-g**) Representative images of human microscopic liver metastasis (**e**), gross liver metastasis (**f**) and primary PDAC tumors (**g**) stained for FSP1 by IHC. (h) Quantification of FSP1+ area per 20X field for gross metastases (n=18) and primary tumors (n=6) or within one cell diameter for micro-metastases (n=57). For large lesions (gross metastases and primary tumors), five fields were averaged together. Bars represent means ± SD; *, p<0.05; **, p<0.01; #, p<0.001; ##, p<0.0001.

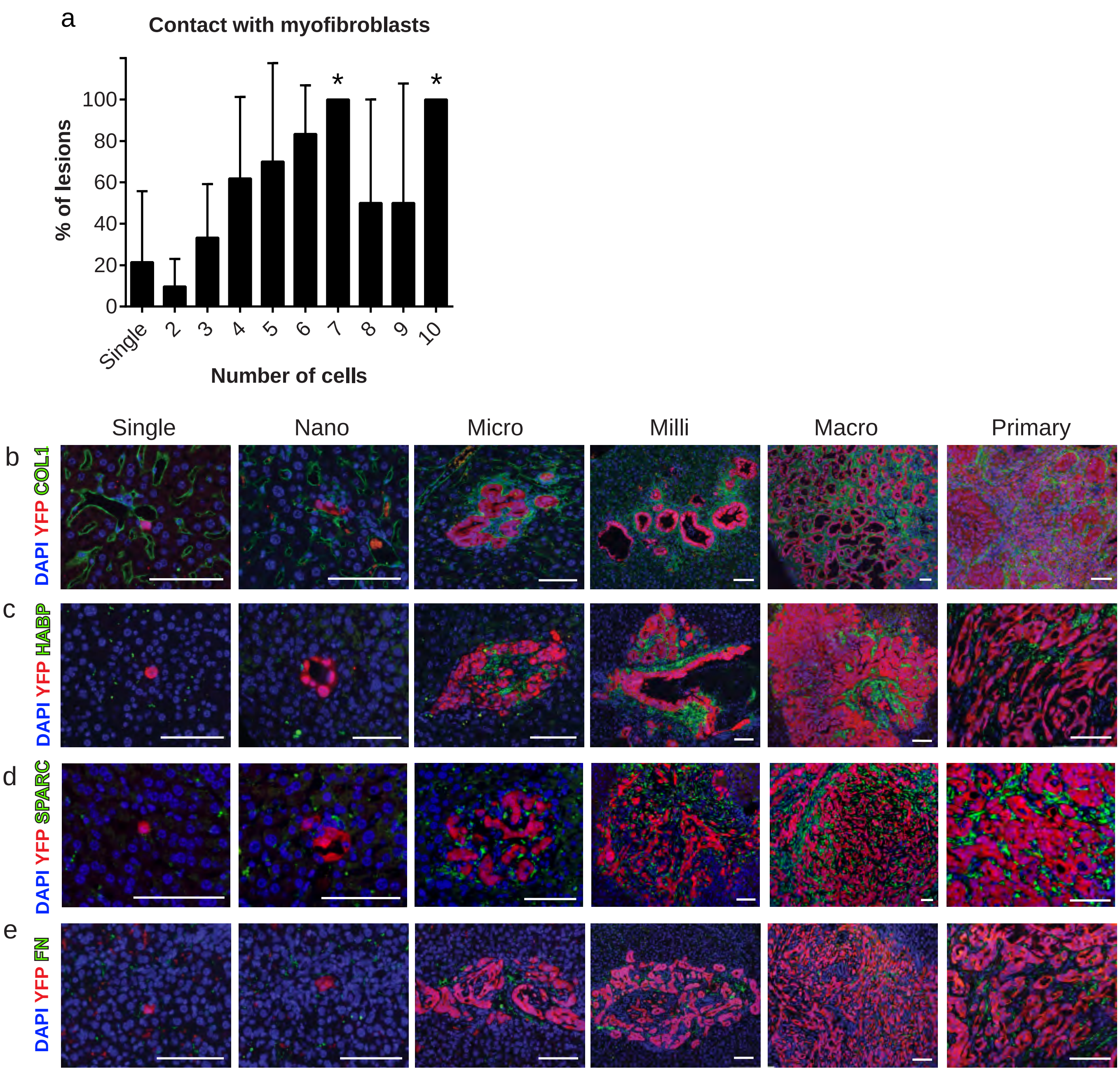

Supplementary Figure 5. Myofibroblast contact with nano-metastases depends on cell size and representative images of ECM stains. (**a**) Contact between nano-metastases and myofibroblasts by cell number. Data are shown as mean ± SD. P values were calculated by one sample t-tests against 'single cell' mean; *, $p<0.05$. (**b-e**) Representative images of primary tumor and metastases stained for YFP (red), DAPI (blue) and (**b**) Collagen 1 (COL1); (**c**) Hyaluronic acid binding protein (HABP); (**d**) Secreted protein acidic and rich in cysteine (SPARC); and (**e**) Fibronectin (FN) in green. Scale bars, 50μm.

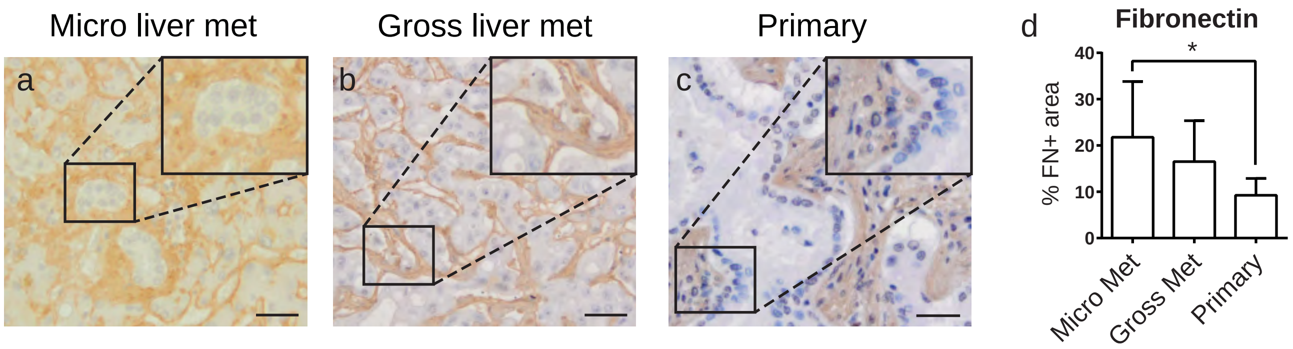

Supplementary Figure 6. Fibronectin deposition in human PDAC tumors and metastases. (a-c) Representative images of human microscopic liver metastasis (n=52), gross liver metastasis (n=18) and primary PDAC tumors (n=6) stained for FN by IHC. (d) Quantification of FN+ area per 20X field for gross metastases and primary tumors or within one cell diameter for micro-metastases. For large lesions (gross metastases and primary tumors), five fields were averaged together. Bars represent means ± SD; *, $p<0.05$.

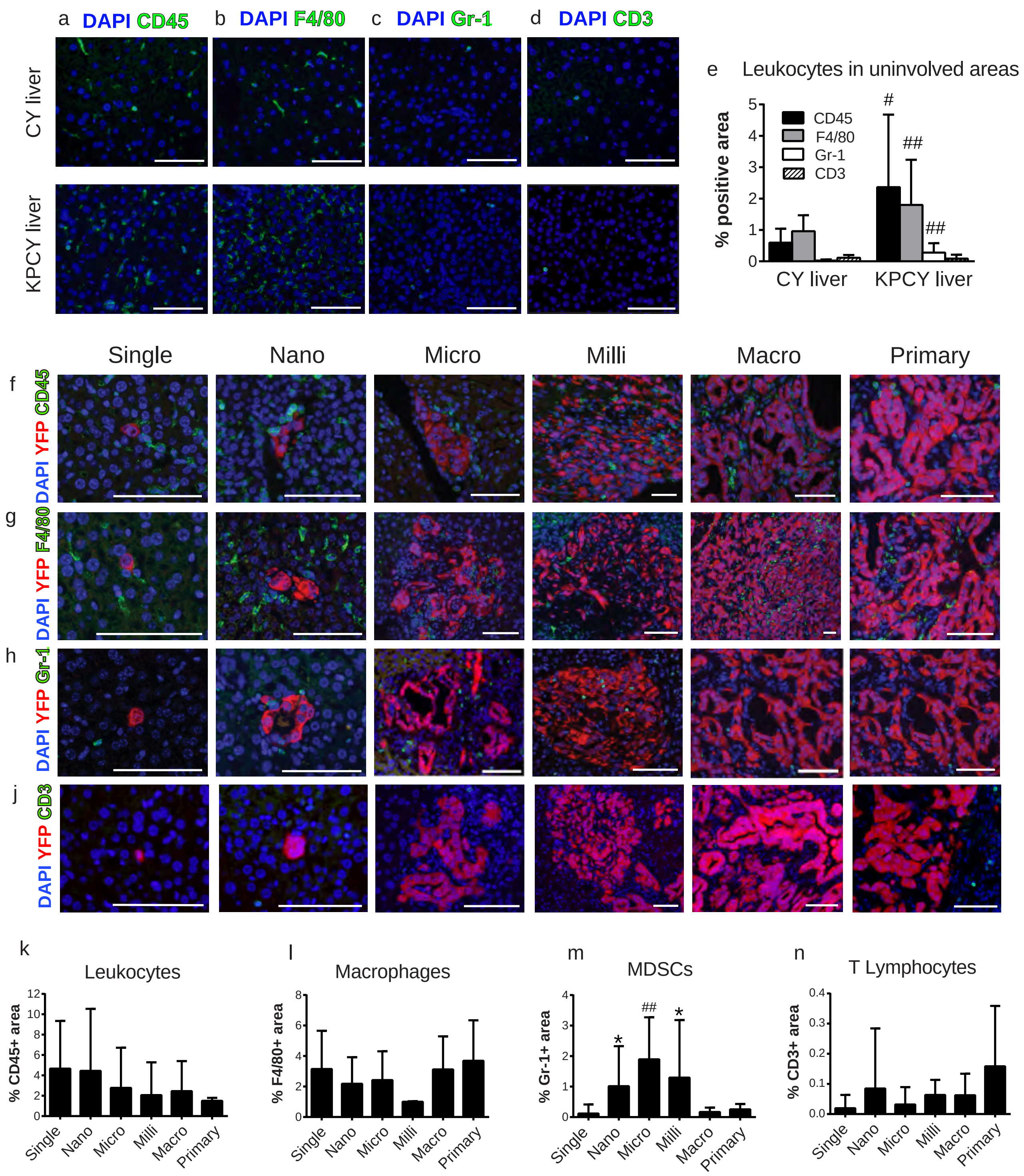

Supplementary Figure 7. Leukocytes accumulate in metastatic livers. (**a-d**) Representative images of CD45 (**a**), F4/80 (**b**), Gr-1 (**c**) and CD3 (**d**) staining (green) and DAPI (blue) in uninvolved areas of control (Pdx1-cre, Rosa$^{Y/Y}$) and metastatic (KPCY) livers. Scale bars, 50µm. (**e**) Quantification of leukocyte density in uninvolved areas of liver (CD45, p=0.0002; F4/80, p<0.0001; Gr-1, p<0.0001; CD3, p=0.3243). (**f-j**) Representative images of leukocyte density at metastatic lesions. Metastases and primary tumor were stained for YFP (red), DAPI (blue) and (**f**), CD45 (leukocytes); (**g**), F4/80 (macrophages); (**h**), Gr-1 (MDSCs); and (**j**), CD3 (T cells) in green. Scale bars, 50µm. (**k-n**) Quantification of leukocyte density at metastatic lesions. The percent positive area for each leukocyte stain was quantified within one cell diameter of metastatic lesions. Data are presented as mean ± SD. Data are presented as mean of the percent positive area within a 40X field ± SD. n ≥ 5 mice, ≥ 50 lesions for each stain. P values were calculated by one-way ANOVA and unpaired Student's t-test with Welch's correction; *, p<0.05; **, p<0.01; #, p<0.001; ##, p<0.0001.

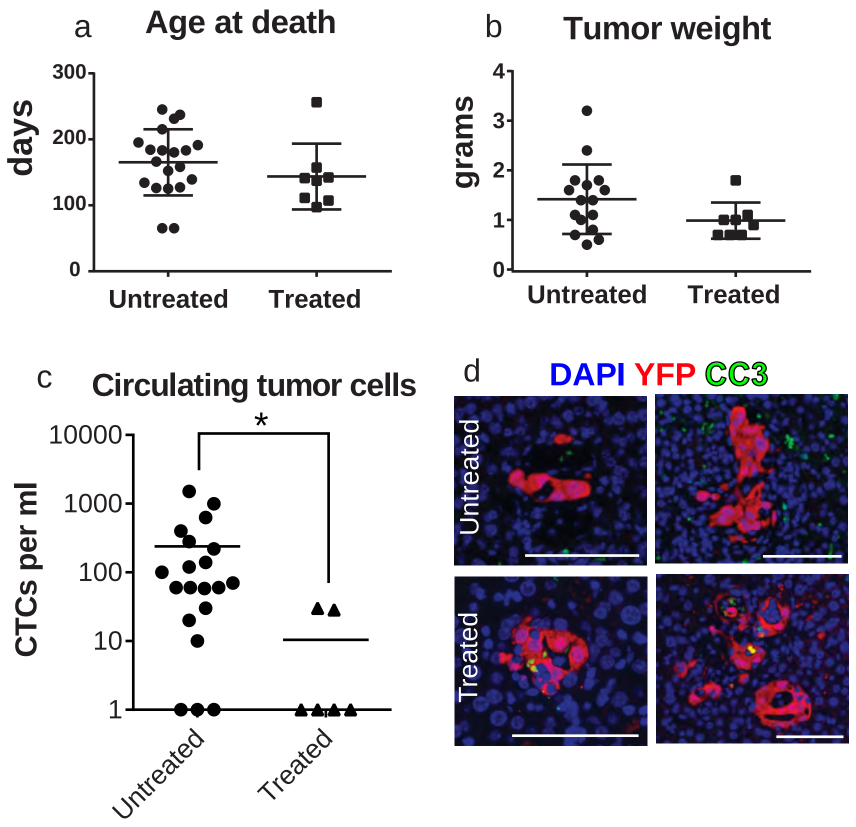

Supplementary Figure 8. Circulating tumor cells (CTCs) are decreased after long-term chemotherapy. (**a-b**) Age at death (**a**) and tumor weight (**b**) for untreated historical controls and treated animals. Lines represent mean ± SD. P value by Student's t-test. (**c**) CTCs in untreated (n=23) and treated (n=8) mice; *, $p < 0.05$. Lines represent the mean. P value by Student's t-test. (**d**) Representative images of untreated and treated metastatic lesions stained for CC3 (green), YFP (red) and DAPI (blue). Scale bars, 50μm.

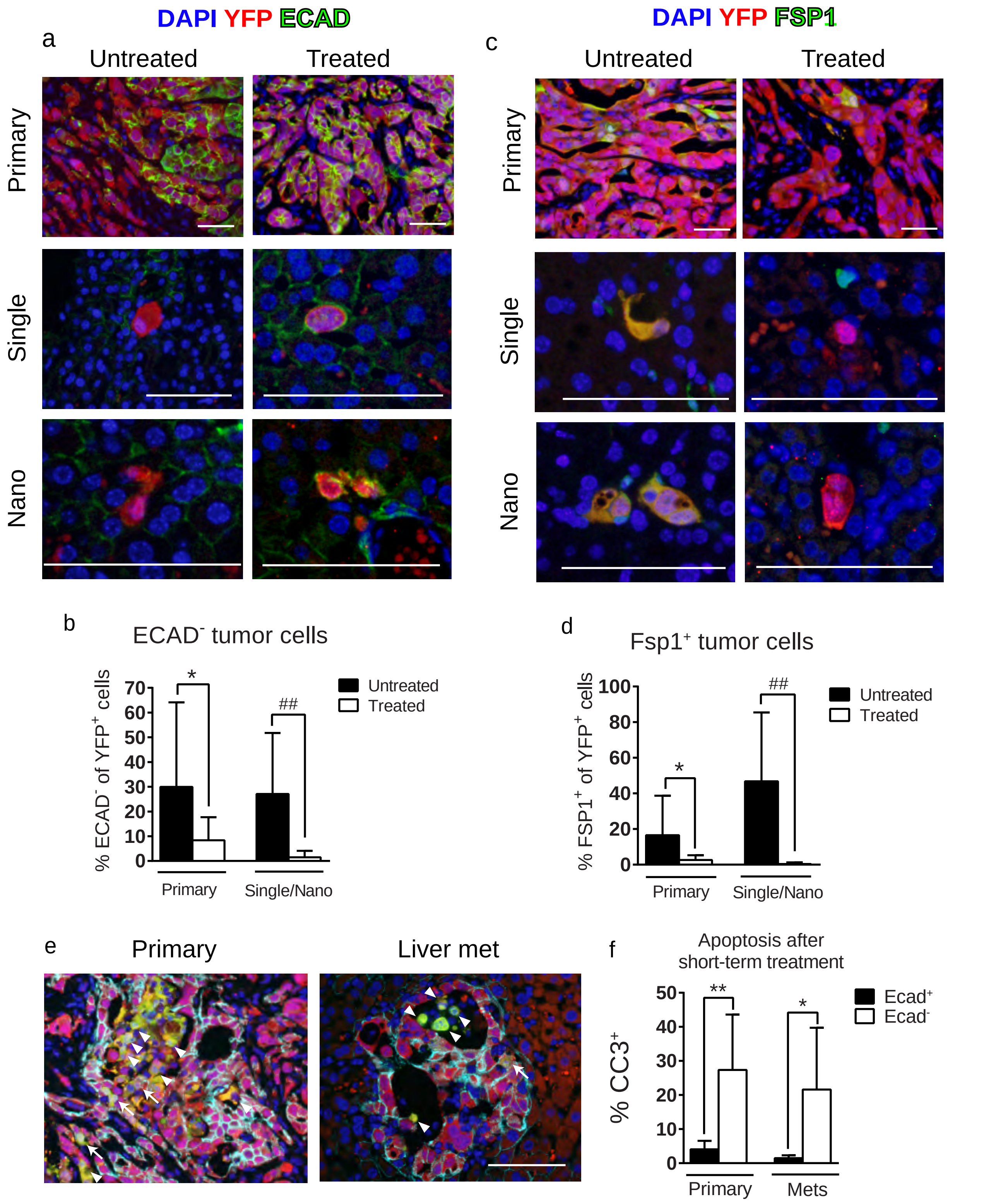

Supplementary Figure 9. Mesenchymal tumor cells are depleted after chemotherapy. (a) Representative images of untreated and treated (GEM/PTX for 2-4 weeks) primary tumors and small metastatic lesions stained for ECAD (green), YFP (red) and DAPI (blue). (b) Quantification of $ECAD^-$ tumor cells in untreated and treated primary tumors and small metastatic lesions. (c) Representative images of untreated and treated primary tumors and small metastatic lesions for FSP1 (green), YFP (red) and DAPI (blue). (d) Quantification of $FSP1^+$ tumor cells in untreated and treated primary tumors and small metastatic lesions. n ≥ 7 mice for each condition. Bars represent means ± SD. Statistical significance was determined by Student's t-test with Welch's correction. (e) Representative images of treated (1 dose GEM/PTX) primary tumor and liver metastasis stained for CC3 (green), ECAD (cyan), YFP (red) and DAPI (blue). Arrows denote $YFP^+/ECAD^+/CC3^+$ cells, arrowheads denote $YFP^+/ECAD^-/CC3^+$ cells. (f) Quantification of CC3 staining in $ECAD^+$ and $ECAD^-$ tumor cells (n ≥ 4 mice). Bars represent means ± SD. Statistical significance was determined by Mann-Whitney test. Scale bars, 50 um. *, p<0.05; **, p<0.01; #, p< 0.001; ##, p < 0.0001.

Mouse ID	Single	Nano	Micro	Milli	Macro	Total
**PD012**	1	1	0	0	0	2
**PD122**	1	1	0	0	0	2
**PD1806**	7	13	19	11	10	60
**PD1821**	9	33	21	1	0	64
**PD1849**	1	9	22	11	7	50
**PD2039**	11	15	5	0	0	31
**PD2204**	14	16	12	0	1	43
**PD2329**	1	0	0	0	0	1
**PD2420**	0	1	6	1	0	8
**PD2523**	13	16	6	0	1	36
**PD333**	1	0	0	0	0	1
**PD350**	24	48	5	0	0	77
**PD422**	6	69	120	41	11	247
**PD431**	12	31	69	27	10	149
**PD463**	5	4	0	0	0	9
**PD483**	1	8	10	5	1	25
**PD487**	5	2	0	0	0	7
**PD635**	25	44	34	10	8	121
**PD661**	10	20	2	0	0	32
**PD796**	3	3	0	0	0	6
**PD798**	4	8	1	0	0	13
**PD8482**	1	3	0	0	0	4
**PD883**	37	94	35	9	3	178

Supplementary Table 1. Metastatic burden (raw counts) for each animal used in Figure 1b.

Antibody	Concentration	Company	Catalog #
Goat and Chicken α GFP	1:500	Abcam	ab6673
Rabbit α CK19	1:1000	In house	N/A
Rabbit α Phospho-Histone H3	1:400	Cell Signaling	D2C8
Rat α Ki67	1:100	DAKO	M7249
Rabbit α PCNA	1:1000	Cell Signaling	D3H8P
Rabbit α Cleaved Caspase-3	1:200	Cell Signaling	9664
Rat α E-cadherin	1:1000	Invitrogen	131900
Rabbit α Claudin-7	1:100	Abcam	ab27487
Rabbit α Fsp1	1:500	DAKO	A5114
Rabbit α Zeb1	1:100	Santa Cruz	sc-25388
Rabbit α αSMA	1:100	Abcam	ab5694
Rabbit α Collagen I	1:200	Abcam	ab34710
Biotinylated bovine hyaluronic acid binding protein	1:200	Calbiochem	385911
Goat α SPARC	1:100	R&D Systems	AF942
Rabbit α Fibronectin	1:300	Abcam	ab2413
Rat α CD45	1:50	BD Biosciences	553076
Rat α F4/80	1:50	Ebioscience	14-4801
Rat α Gr-1	1:50	Ebioscience	14-5931
Rat α CD3	1:50	Ebioscience	14-0032
Goat α VE-cadherin	1:100	R&D Systems	AF1002

Supplementary Table 2. Antibodies used for immunofluorescence and IHC.

**Average age ± SD**	61.6 ± 11.3	**Chemotherapy, n (%)**	23 (76.7)
**Average OS, mo ± SD**	10.5 ± 9.2	**Radiation, n (%)**	10 (33.3)
**Average tumor size, cm ± SD**	5.6 ± 3.0	**Metastatic burden, n (%)**	
**Sex, n (%)**		≤10	2 (6.7)
Male	18 (60)	11-100	10 (33.3)
Female	12 (40)	>100	18 (60)
**Location, n (%)**		**Differentation, n (%)**	
Head	18 (60)	Well/Moderate	1 (3.3)
Body	7 (23.3)	Moderate	10 (33.3)
Tail	4 (13.3)	Moderate/Poor	12 (40)
**Unknown, n (%)**	1 (3.4)	Poor	6 (20)
**Stage at diagnosis, n (%)**		**KRAS status, n (%)**	
IIB	2 (6.7)	Mutant	29 (96.7)
III	7 (23.3)	WT	1 (3.3)
IV	21 (70)	**TP53 status, n (%)**	
**Surgery, n (%)**	4 (13.3)	Mutant	26 (86.7)
		WT	4 (13.3)

Supplementary Table 3. Clinicopathological characteristics of patients. Abbreviations: standard deviation (SD); overall survival (OS); month (mo); number of patients (n); wildtype (WT).
